# Species-Specific Viromes in the Ancestral Holobiont *Hydra*

**DOI:** 10.1371/journal.pone.0109952

**Published:** 2014-10-24

**Authors:** Juris A. Grasis, Tim Lachnit, Friederike Anton-Erxleben, Yan Wei Lim, Robert Schmieder, Sebastian Fraune, Sören Franzenburg, Santiago Insua, GloriaMay Machado, Matthew Haynes, Mark Little, Robert Kimble, Philip Rosenstiel, Forest L. Rohwer, Thomas C. G. Bosch

**Affiliations:** 1 Department of Biology, San Diego State University, San Diego, California, United States of America; 2 Zoological Institute, Christian-Albrechts University Kiel, Kiel, Germany; 3 Institute of Clinical Molecular Biology, Christian-Albrechts University Kiel, Kiel, Germany; University of North Carolina at Chapel Hill, United States of America

## Abstract

Recent evidence showing host specificity of colonizing bacteria supports the view that multicellular organisms are holobionts comprised of the macroscopic host in synergistic interdependence with a heterogeneous and host-specific microbial community. Whereas host-bacteria interactions have been extensively investigated, comparatively little is known about host-virus interactions and viral contribution to the holobiont. We sought to determine the viral communities associating with different *Hydra* species, whether these viral communities were altered with environmental stress, and whether these viruses affect the *Hydra*-associated holobiont. Here we show that each species of *Hydra* harbors a diverse host-associated virome. Primary viral families associated with *Hydra* are *Myoviridae*, *Siphoviridae*, *Inoviridae*, and *Herpesviridae*. Most *Hydra*-associated viruses are bacteriophages, a reflection of their involvement in the holobiont. Changes in environmental conditions alter the associated virome, increase viral diversity, and affect the metabolism of the holobiont. The specificity and dynamics of the virome point to potential viral involvement in regulating microbial associations in the *Hydra* holobiont. While viruses are generally regarded as pathogenic agents, our study suggests an evolutionary conserved ability of viruses to function as holobiont regulators and, therefore, constitutes an emerging paradigm shift in host-microbe interactions.

## Introduction

There has been tremendous progress during the past two decades in elucidating general principles of host-bacteria interactions and the factors that control them [1], [2]. Host-bacteria interactions have been studied in a variety of animals indicating that the evolution of the microbiome can be at least traced to the base of metazoan life [3], [4]. Consistent with this view, early studies in the eumetazoan phylum Cnidaria suggest that these animals live in constant interaction with a microbial surrounding [5], [6]. More recent experiments with the Cnidarian *Hydra* showed that the colonizing bacteria are species-specific and that *Hydra* share a mutualistic relationship with their microbiota [7], [8]. *H. magnipapillata* and *H. vulgaris*, for example, each associate with different populations of β-proteobacteria, bacteroidetes, and γ-proteobacteria, while *H. oligactis* primarily associates with 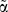 proteobacteria, and *H. carnea* related laboratory strain *H. vulgaris* (AEP) associates with β-proteobacteria and some 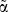 proteobacteria [9], [10]. *H. viridissima* mostly associates with an *Alcaligenaceae* bacterial population [10]. These experiments and subsequent studies led to the view that the host epithelium actively selects for its resident microbiota and that the microbiota is a complex trait that is under strong host genetic control [10], [11], [12]. This assemblage of host in symbiosis with its associated microbes is called a holobiont [13], [14].

Although viruses are the most abundant and diverse biological component on the planet, comparatively little is known about their role as an integral part of the holobiont. Viruses are found anywhere cellular life exists, in any environment or ecosystem. They exist along with their hosts as part of a dynamic community ensemble of exogenous viral particles and endogenous proviruses [15]. Viruses are vital to evolution, to pathogenesis, and to metabolic cycles [16]. Since the majority of viruses cannot be propagated through culturing, understanding their role in the evolution of the holobiont and in controlling the bacterial composition has resisted analysis [17]. We therefore sought to determine the presence of viruses associating with animals at one of the earliest stages of metazoan evolution, to characterize the communities associating with these basal animals, so that we may better understand viral standing within the holobiont.

Here we use the model host *Hydra* and culture-independent methods to investigate the unknown specificity and dynamics of the viral flora in one of the simplest animals at the tissue level of organization. The results show that each analyzed *Hydra* species is associated with a diverse DNA viral metagenome (virome) indicating the existence of a complex viral community present in organisms at the base of animal evolution. This supports findings that these animals associate with diverse bacterial communities [7], [8]. The observation that the majority of viruses associating with different *Hydra* species are bacteriophages points to a potential impact of the virome on the evolution and maintenance of the *Hydra* holobiont.

## Materials and Methods

### *Hydra* Species

*Hydra magnipapillata*, *H. oligactis*, *H. viridissima*, and *H. vulgaris* (AEP) were all kept at 18°C and fed a standardized artemia diet. Wild-caught *H. vulgaris* were collected from Santee Lake in San Diego County (32.50′46″N 117.00′17″W) in January 2012.

### Sampling Procedure, Viral Purification, Nucleic Acid Extraction and Sequencing

Approximately 100 animals were sampled for each virome (∼100 mg). The animals were separated into a dish containing either 0.45-µm filtered and autoclaved *Hydra* medium (for laboratory reared animals), or 0.45-µm filtered and autoclaved lake water (for wild-caught animals), and were not fed for 2 days prior to processing. In the intervening days, the animal cultures were washed with 0.45-µm filtered and autoclaved *Hydra* medium or 0.45-µm filtered and autoclaved lake water to ensure no other eukaryotic organisms were present. Approximately 100 animals each were placed in a 15 ml Falcon tube. One tube was kept at 18°C for 24 hours (non-stressed animals); the other tube was kept in a 23°C water bath for 24 hours prior to homogenization (heat-stressed animals, 5°C above non-stressed animals). Animals were transferred to a microcentrifuge tube, washed twice with 1 ml virus-free 0.02-µm filtered Milli-Q dH_2_O, resuspended in 1 ml virus-free 0.02-µm filtered Milli-Q dH_2_O, and then homogenized by pestle until no particulate matter could be visualized. The animals at this point had been free of prey for a minimum of 72 hours. The homogenate was centrifuged at 4500-x g for 5 minutes to pellet cellular debris. The supernatant was filtered through a 0.45-µm filter to remove eukaryotic cellular components while preserving larger viruses. The sample was then DNase-treated (2.5 U/ml) for 1 hour at 37°C, and the reaction was stopped by incubation at 65°C for 10 minutes. A subsample was taken to verify virus-like particle (VLP) presence by filtering onto a 0.02-µm Anodisc polycarbonate filter (Whatman, GE Healthcare Life Sciences, Freiburg, Germany). Filters were stained with 5× SYBR Gold, washed and visualized on an epifluorescent microscope. More than 30 images per sample were taken to determine the VLP to bacteria ratio. Images were analyzed by ImagePro Plus 5.1 software. The DNase-treated 0.45-µm filter eluates were then treated with 0.2 volumes chloroform, centrifuged at 13,800×g for 15 minutes, and the supernatant was transferred to a clean microcentrifuge tube. Another subsample was taken for transmission electron microscopy to verify virus morphotypes. This subsample was absorbed on a 300-mesh carbon-formar grid, negatively stained with 2% uranyl acetate and visualized using a transmission electron microscope at 40,000X. Filtered, DNase-treated and chloroform-treated samples were then placed on a CsCl density gradient and ultracentrifuged at 62,000-x g for two hours. The viral fraction was collected and again treated with 0.2 volumes chloroform and DNase, as described above. The purified viral sample DNA was then extracted using a formamide, cetyltrimethylammonium bromide, phenol chloroform procedure detailed in Thurber, et al [18]. To eliminate the possibility of prokaryotic or eukaryotic contamination in our sequenced viromes, purified viral DNA was determined to be both 16S and 18S rRNA gene negative by PCR prior to sequencing. Since little viral DNA was present (approximately 20–200 ng per pooled sample), viral DNA was amplified using an illustra GenomiPhi V2 DNA amplification kit (GE Healthcare Life Sciences, Freiburg, Germany) to obtain ∼1 µg of viral DNA for sequencing. Further, to ensure sample processing was not contaminated, we tried to amplify a reaction without template using Phi29, but were unable to amplify enough DNA to construct a library for sequencing. To minimize bias of circular genomes and single-stranded viruses by Phi29 DNA polymerase, the amplification reaction was done in quadruplicate, pooled, ethanol purified, and then pyrosequenced using a Roche 454 Life Sciences GS FLX Titanium platform (Roche 454 Life Sciences, Basel, Switzerland). It should be noted that there are concerns for using pooled MDA reactions [19], and that this method should be avoided to minimize sample variation in future experiments by using replicate samples.

### Taxonomic and Functional Annotations

Pyrosequencing multiplex identifiers (MIDs) were removed, minimum read lengths (<60 bp) were removed, quality read scores of <20 were removed, reads with>1% ambiguous bases were removed, low complexity reads (entropy>70) were removed and duplicate reads were all removed using the programs TagCleaner [20] and PRINSEQ [21]. DeconSeq removed contaminant sequences (coverage>90, identity>90) from Chordates, Cnidarian, and Vectors [22]. Only sequences devoid of Chordate, Cnidarian, and vectors sequences were analyzed. Additionally, we searched for Arthropod sequences in the sequenced removed by DeconSeq and found no sequences in these rejected sequences indicating little to no prey contamination. Metagenome sequences were queried by BLASTN using an E-value threshold of ≤10^−5^ to the Genbank non-redundant nucleotide database. Metagenome sequences were normalized using GAAS [23] and taxonomies were assigned according to best similarities. Sequence similarities to known viral genomes were resolved by TBLASTX comparison to a NCBI viral database at an E-value threshold of ≤10^−5^. PHACCS (v. 1.13) was used for each virome to determine the diversity of each viral community [24]. The diversity was predicted according to the contig spectrum and average read length for each virome. All other parameters were set in the default position. Rarefaction analyses of the metagenomes were performed through MG-RAST to verify the sequencing of sufficient gene clusters for the viral communities associated with each *Hydra* species community sample.

### Viral Genome Assemblies

*De novo* assembly of the viral genomes was performed using MIRA software (v.3.4) with a minimum length overlap of ≥35 bp and minimum overlay identity of ≥98%. Only contigs longer than 500 bp were kept. Assemblies were additionally queried by TBLASTX using an E-value threshold of ≤10^−5^ to a NCBI viral database to identify the species of viral genomes within the assemblies. Assemblies were visualized through use of the contig map feature on Metavir [25]. Cross-assembly of the viral metagenomes was performed by concatenating all of the sequences from the 10 viromes into one file, performing *de novo* assembly on this file using MIRA, and then comparing each of the individual virome sequences to the cross-assembly using the program crAss [26]. The individual viromes compared to the full assembly were visualized using the distance formula “Wootters” and resulting cladogram from the crAss output.

### System Metabolic Potential

Each of the assembled viral metagenomes was submitted to MG-RAST for comparison by TBLASTX to the metabolic subsystem SEED database at an E-value threshold of ≤10^−5^ [27]. The potential for each virome to affect the metabolism of a holobiont community was determined by assigning functional annotations to the sequences, and then assigning the sequences to a specific biological process. Comparisons of each *Hydra* species virome subsystem were made in a pairwise manner (non-stressed v. heat-stressed) using XIPE-Totec nonparametric statistical analysis [28]. Analyses were conducted under 95% confidence and 500 iterations.

### Nucleotide Sequence Accession Numbers

Viral metagenomes are freely available on the MG-RAST annotation server with the following accession numbers: *H. vulgaris* wild-caught non-stressed (4491643.3) and heat-stressed (4491646.3), *H. vulgaris* (AEP) non-stressed (4491638.3) and heat-stressed (4491639.3), *H. magnipapillata* non-stressed (4491640.3) and heat-stressed (4491641.3), *H. oligactis* non-stressed (4491635.3) and heat-stressed (4491637.3), and *H. viridissima* non-stressed (4491642.3) and heat-stressed (4491645.3).

## Results

### Identification and characterization of the *Hydra* virome

Viruses were isolated from five different species of *Hydra*, encompassing each of the major taxonomic groups of these animals. This includes *H. vulgaris* collected from a lake in San Diego County, the genome sequenced *H. magnipapillata*, *H. oligactis*, the zoochlorellae endosymbiont containing *H. viridissima*, and the common laboratory strain *H. vulgaris* (AEP). *H. vulgaris* (AEP) strain of *Hydra* is more genetically related to *H*. *carnea* and associates with a distinct microbial community, differentiating this strain from the wild-caught *H*. *vulgaris* [7], [29]. The animals were starved for 48 hours to reduce potential viral background deriving from the prey. Then one set of animals from each species was kept at ambient temperature (18°C), while another set was heat-stressed at 23°C for 24 hours to induce environmental stress. Approximately 100 individuals per species and treatment were used for viral extraction. Viral to bacterial ratios were evaluated by epifluorescence microscopy. Purified viruses were tested for host and bacterial contamination by PCR; negative of both prokaryotic (16S) and eukaryotic (18S) rRNA genes. Viral DNA was extracted, random hexamer amplified by Phi29 polymerase and sequenced through use of 454 pyrosequencing.

The virus-like particle (VLP) to bacteria ratio for non-stressed *H. vulgaris* (AEP) was found to be statistically different from that of heat-stressed *H. vulgaris* (AEP) (Figure S1; non-stressed VLP: Bacteria 4.4±0.6; heat-stressed VLP: Bacteria 2.3±0.3; Student's t-test p = 0.002). Similar to published viral metagenomes, most of the sequences did not match any known sequences, accounting for 50–87% of the sequences, as determined by BLASTN comparison of a non-redundant database at an E-value threshold of ≤10^−5^ (Table 1). Of the ten viromes, the percentage of sequence similarities to known viral genomes ranged from 4–33%, as resolved by TBLASTX comparison to a NCBI viral database at an E-value threshold of ≤10^−5^. This is similar to published viral metagenomes [30]–[35], and an improvement with respect to the percentage of viral hits in the wild-caught *H. vulgaris* viromes (non-stressed, 28.4%; heat-stressed, 33.9%). Rarefaction analysis was performed to determine whether each of the 10 viromes of more than 100-pooled animals were sequenced to the point of saturation as assessed by gene clusters and found to be similar to other published virome studies (Figure S2). Further, diversity estimations of the viromes were performed using PHACCS [24]. PHACCS performs statistical analyses of the richness, evenness, diversity, and abundance of the viral communities from the metagenomic reads. The sample with the highest viral diversity was heat-stressed *H. magnipapillata*, as determined by viral species richness (Table 1). Most *Hydra*-associated viral communities had equally abundant genotypes, as assessed by evenness approaching 1 (Table 1). Finally, the Shannon-Wiener index shows that viral diversity increased in each *Hydra* species with heat-stress, which supports the impact of heat stress on the host-associated viral community (Table 1).

**Table 1:** Virome Sequencing Characterization.

	*H. vulgaris* Wild-Caught		*H. oligactis*		*H. magnipapillata*		*H. viridissima*		*H. vulgaris* (AEP)	
Heat-Stress:	-	+	-	+	-	+	-	+	-	+
**Sequences** *	44726	20855	47968	59847	2159	47709	37391	21304	40104	70555
**Avg. Seq. Length (bp)**	352	296	391	389	471	440	329	251	410	351
**Unknown Sequences (% of Seq.)**	22813 (51.0)	13476 (64.6)	37041 (77.2)	45625 (76.2)	1309 (60.6)	41364 (86.7)	18990 (50.8)	11108 (52.1)	29460 (73.5)	61673 (87.4)
**Known Sequences (% of Seq.)**	21913 (49.0)	7139 (34.2)	10926 (22.8)	14222 (23.8)	850 (39.4)	6345 (13.3)	18401 (49.2)	10196 (47.9)	10644 (26.5)	8882 (12.6)
**Viral Sequences (% of Seq.)**	12713 (28.4)	6907 (33.9)	5036 (10.5)	6567 (11.0)	236 (10.9)	2003 (4.2)	5580 (14.9)	5566 (26.1)	7284 (18.2)	6503 (9.2)
**Prokaryotic Viral Sequences (% of Seq.)**	6354 (14.2)	3318 (15.9)	3227 (6.7)	3943 (6.6)	100 (4.6)	768 (1.6)	2522 (6.8)	2778 (13.0)	4224 (10.5)	2610 (3.7)
**Eukaryotic Viral Sequences (% of Seq.)**	6327 (14.1)	3577 (17.2)	1784 (3.7)	2582 (4.3)	134 (6.2)	1210 (2.5)	3019 (8.1)	2756 (12.9)	2952 (18.1)	3783 (5.4)
**Richness**	24	85	694	1135	29	6328	56	72	2000	2189
**Evenness**	0.92	0.96	0.85	0.93	0.89	0.86	0.97	0.97	0.55	0.80
**Most Abundant Genotype %**	18.03	6.09	7.96	7.14	18.88	4.11	5.57	6.56	25.33	8.50
**S-W Index (Nats)**	2.91	4.27	4.57	5.59	3.01	7.56	3.91	4.16	4.17	6.17

*Total sequence reads after contaminant sequence reads were removed by DeconSeq program.

The phylogenetic profiles of the ten viromes were initially compared to that of published viromes from animal and water environments (Figure S3). Comparison through the MG-RAST server against a SEED database and visualization through principal component analysis (PCoA) revealed that the *Hydra* viromes compared favorably to coral and fish slime viromes but not as well to human or fish gut animal viromes (Figure S3A) [30], [31], [32]. The *Hydra* viromes also compared favorably to temperate freshwater pond viromes while not as well to hotter (Hot Springs) or colder (Antarctic Lake) freshwater environment viromes (Figure S3B) [33], [34], [35]. These data indicate that the *Hydra* host associates with viromes similar to those found within exposed epithelium in temperate freshwater environments.

**Figure 1:**
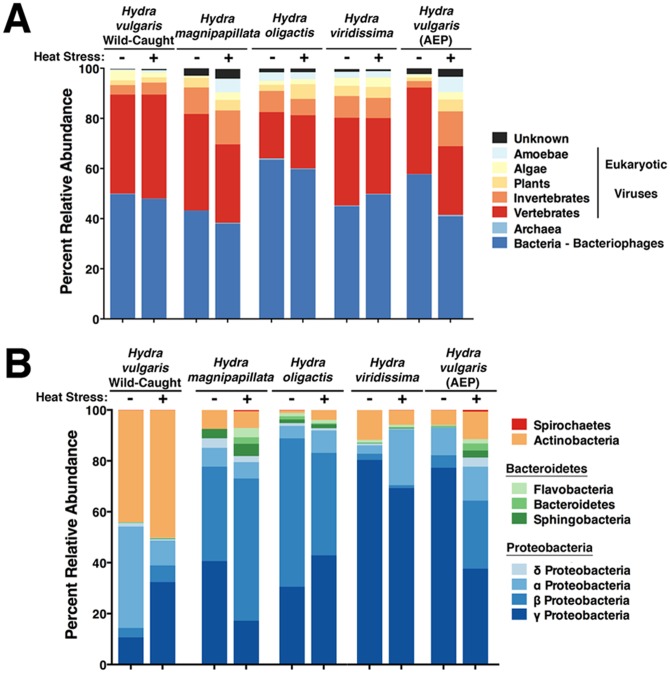
The relative abundance of viral types and the bacterial class hosts of associating viruses to various *Hydra* species. A) Predicted host range of each *Hydra* associated virome, including eukaryotic and prokaryotic viruses. B) Predicted bacterial host range for the bacteriophages associating with *Hydra* species. The order in each column is equivalent to the order in the legend.

Figure 1A displays the results of the types of viruses associated with each species of *Hydra* under both non-stressed and heat-stressed conditions. The largest percentages of associating viruses for each species of *Hydra* are bacteriophages, or viruses that infect prokaryotes, both in the bacterial and archaeal domains. Predicted prokaryotic hosts accounted for 38–63% of the viral sequencing hits. Of additional interest are large populations of vertebrate-infecting eukaryotic viruses found associating with each *Hydra* species, accounting for 18–43% of the total viral sequences. Smaller populations of invertebrate-infecting viruses were found, as well as even smaller populations of viruses known to infect algal and plant hosts. Because bacteriophage sequences include bacterial sequences, we were able to identify bacterial taxa and predict the host range of bacteria infected by the *Hydra*-associated bacteriophages. We found that the predicted bacterial host range of these bacteriophages consisted primarily of proteobacteria, particularly that of the γ- and β-proteobacteria classes (Figure 1B). Laboratory-housed polyps host 71–89% of these bacteriophages that infect these classes of proteobacteria. In contrast, wild-caught *H. vulgaris* displayed a distinct community of predicted bacteriophage hosts consisting primarily of actinobacteria and α-proteobacteria hosts.

### *Hydra*-Associated Viruses are Species Specific and Sensitive to Temperature Stress

**Figure 2:**
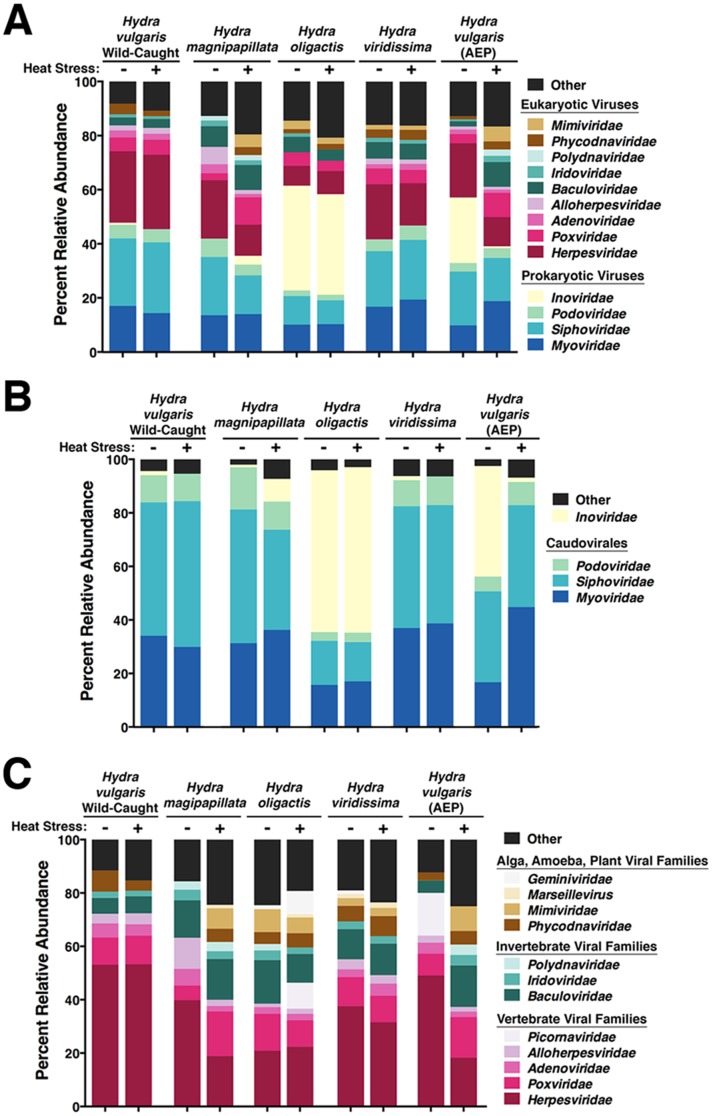
The *Hydra* viromes. The relative abundance and diversity of viral families associating with *Hydra* species in non-stressed and heat-stressed conditions reveals that each species of *Hydra* associates with a unique community of viruses. Results display the relative abundances of families of identified viruses (TBLASTX, E-value threshold ≤10^−5^) associating with the different species of *Hydra* under both non-stressed and heat-stressed conditions for all viral families (A), for prokaryotic viruses (B), and for eukaryotic viruses (C). The order of the viral family in each column is equivalent to the order of the viral family in the legend.

Taxonomic evaluation of the viral families isolated from lab-cultured or wild-caught *Hydra* reveal that each species of *Hydra* associate with a diverse community of prokaryotic and eukaryotic viruses (Figure 2A). The most common viral families associating with *Hydra* are the Caudovirales bacteriophages *Myoviridae*, *Siphoviridae*, and *Inoviridae*, as well as the eukaryotic *Herpesviridae* family of viruses. These four families of viruses accounted for 43–74% of the viral sequences found to associate with the various *Hydra* species. Outside of these families, a wide diversity of viruses unique to each species of *Hydra* are observed. It should be noted that in order to get an overview of the viral communities associated with different species of *Hydra*, each virome analyzed originates from a pooled sample of at least 100 polyps. This is due to insufficient numbers of viruses and/or viral DNA associated with a single polyp needed for sequencing. Therefore these viromes reflect the viruses associating with each *Hydra* species population and are not due to stochastic sampling effects of individuals. Further, since pooled *Hydra* species populations associate with diverse viral communities, this may exhibit greater specificity than when evaluated at the individual level.

Figure 2B shows that the two most common families of bacteriophages associating with most *Hydra* species are the *Myoviridae* and *Siphoviridae* families, accounting for 15–45% and 15–55% of the total number of bacteriophages, respectively. In the cases of *H. oligactis* and non-stressed *H. vulgaris* (AEP) strains, the *Inoviridae* family of bacteriophages is the dominant population of associating prokaryotic viruses. The associated *Podoviridae* bacteriophage family abundance for all the *Hydra* species was frequently less than 10%. Remarkably, only these four bacteriophage families were often associated with *Hydra* species, as no other family made up even 1% of the total bacteriophage population in any of the viromes.

Figure 2C reveals that *Hydra* maintain a diverse community of eukaryotic viruses dominated by a population of *Herpesviridae* family viruses. The *Herpesviridae* family accounts for 18–53% of the total population of associating eukaryotic viruses in *Hydra* species. Interestingly, wild-caught *H. vulgaris* display the largest population of *Herpesviridae* viruses, accounting for 53% of the eukaryotic viruses. Other eukaryotic viral families observed include the *Poxviridae* (infecting vertebrates and invertebrates), *Mimiviridae* (infecting amoebae and algae), and *Phycodnaviridae* (infecting algae).

**Figure 3:**
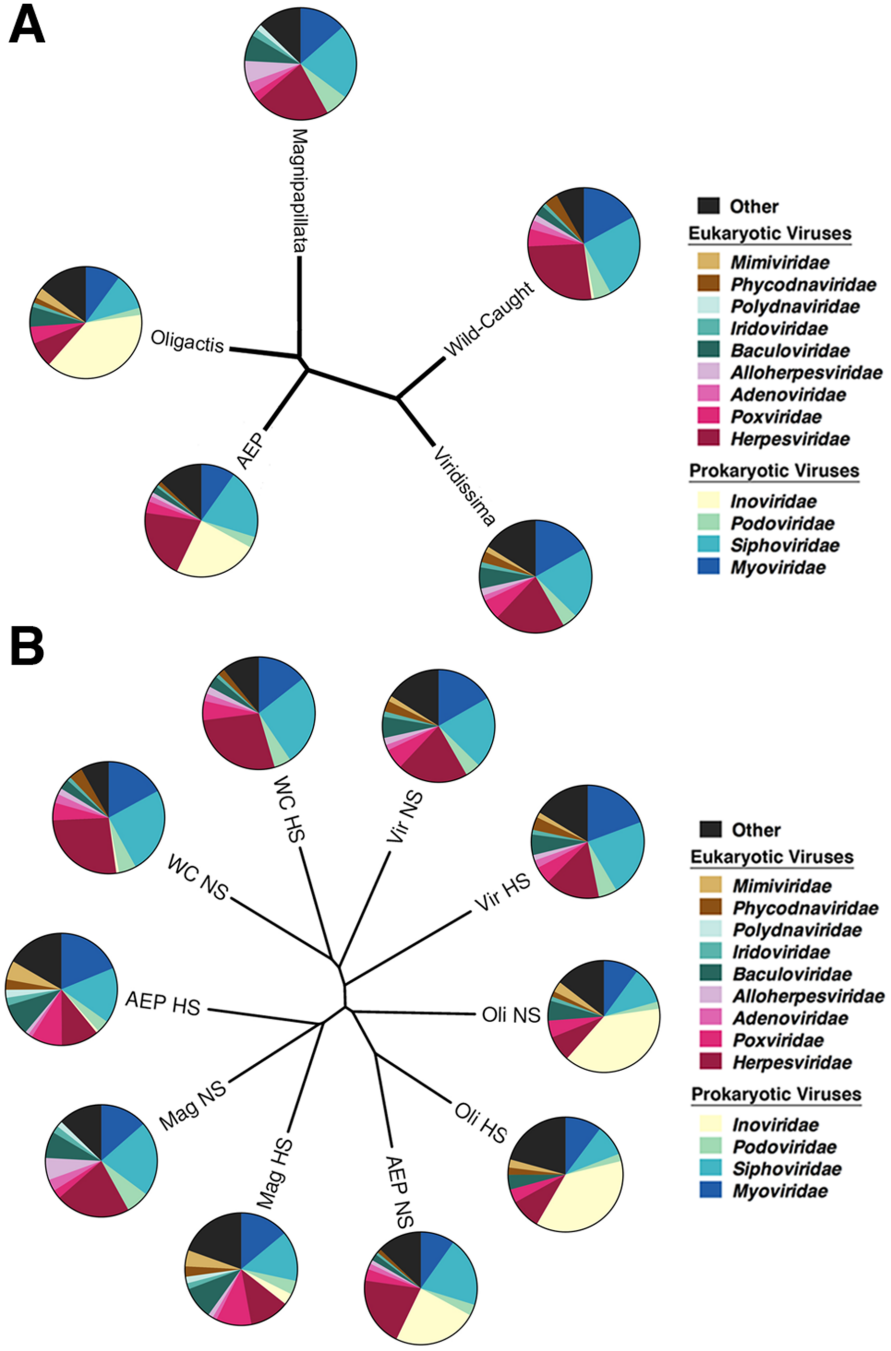
*Hydra* species harbor specific viral communities. Viromes were cross-assembled into one file using MIRA and then each virome was compared to the cross-assembled file using crAss. A) CrAss Wootters formula cladogram output of each *Hydra* species virome. B) CrAss Wootters formula cladogram output of each *Hydra* species under non-stressed (NS) and heat-stressed (HS) conditions. The viral family in each pie chart is listed in the legend.

To determine whether each of the *Hydra* species harbor a specific viral community associating with them, we assessed the metagenomic reads by the crAss program [26]. This program determines the viral community structure from metagenomes by using cross-assembly of reads from different metagenomes to assess the similarities between the sampled communities. All of the viromes were first cross-assembled into one file using MIRA, and then each of the individual viromes were compared to the cross-assembled file using crAss to create a cladogram of the similarities between the viromes (Figure 3A). The cladogram shows very distinct viral populations associating with each of the *Hydra* species.

One additional question we sought to address was the impact of the environment on *Hydra* association with viral communities. To address the extent to which the various viromes were affected during stress, we exposed the host polyps to an elevated culture temperature for 24 hours. In all investigated *Hydra* species, heat-stress induced increase in the diversity of viral families associated with the animal, as assessed by Shannon-Wiener Index (Table 1; Student's t-test; n = 5; F = 8; p<0.05). This was reflected by the induction of large numbers of eukaryotic viruses whose family abundance accounts for less than one percent of the total population, which is classified as “other”. *H. vulgaris* (AEP) displayed an additional alteration of its primary family of viruses, *Inoviridae*, decreasing from 24% of its total virus population in non-stressed conditions to less than one percent upon heat-stress (Figure 2A). Moreover, environmental heat-stress caused each *Hydra* species to alter the viral communities associating with them as determined by the “Wootters” formula cladogram output from the cross-assembly (Figure 3B). Interestingly, in each species, heat-stress also caused the diversity of bacteriophage hosts to increase (Figure 1B). The bacteriophage viromes, however, remain relatively unchanged upon heat-stress, with only the *H. vulgaris* (AEP) strain displaying a reduction in its primary associating bacteriophage family of *Inoviridae* to two distinct populations of *Myoviridae* and *Siphoviridae* (Figure 2B). Taken together, the findings show that environmental stress causes a shift in the community of viruses associated with each *Hydra* species, particularly amongst the eukaryotic viral population.

### Visualization of *Hydra*-Associated Viral Family Members by TEM

**Figure 4:**
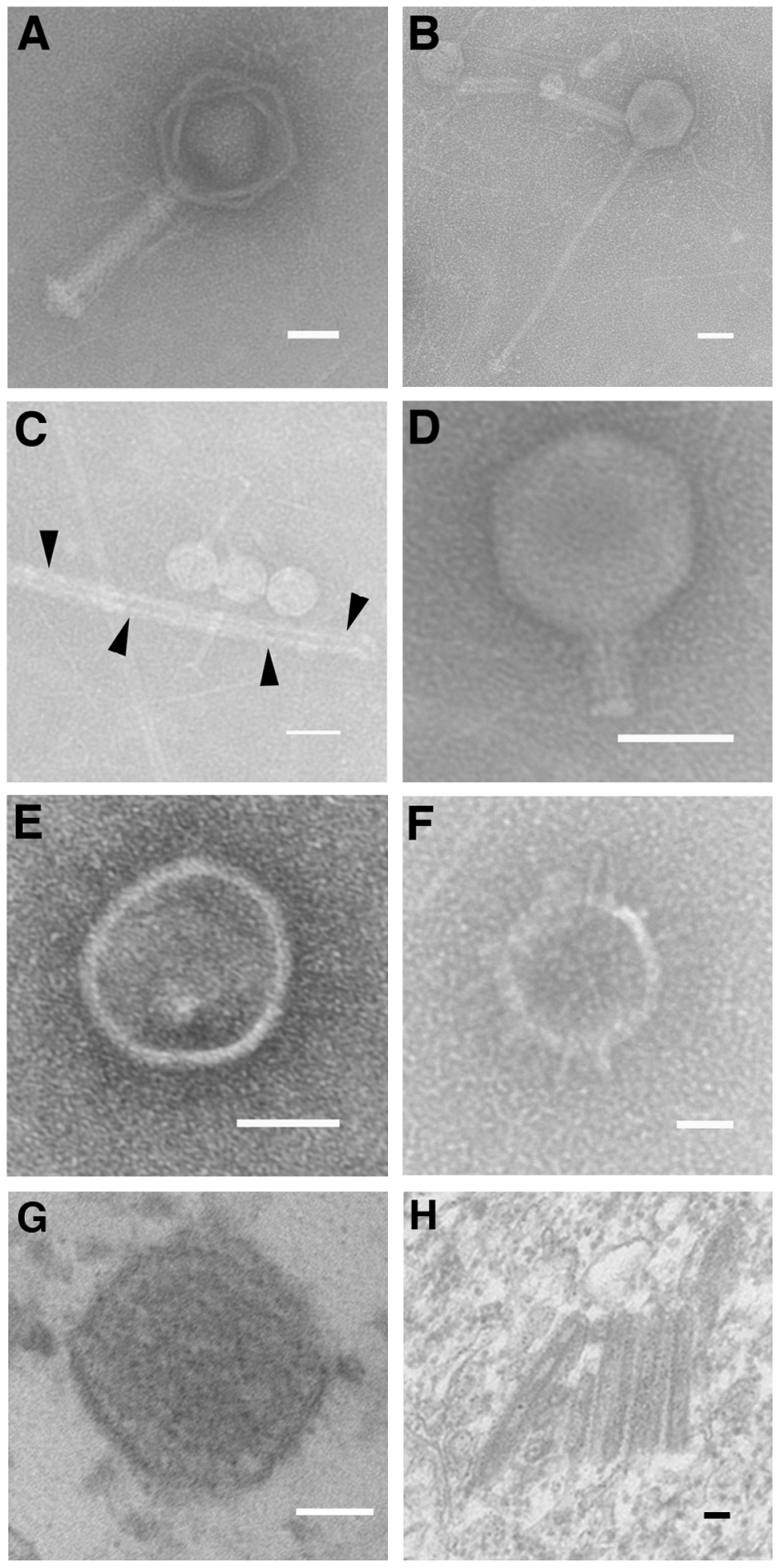
Examples of viral families found in the isolation of viruses from Hydra species. After viral isolation, a subsample of viruses were absorbed on a carbon-formar TEM grid, negatively stained with 2% uranyl acetate, and visualized by transmission electron microscopy at 40000X. Many viral families have been identified, including bacteriophages A) *Myoviridae*, B) *Siphoviridae*, C) *Inoviridae* (arrows point towards *Inoviridae* virion), and D) *Podoviridae*, as well as eukaryotic viral families E) *Herpesviridae*, and F) *Phycodnaviridae*. Tissue bound G) *Herpesviridae* and H) *Baculoviridae*. The bar in each panel is 50 nm.

To visualize the members of the *Hydra* specific viral families and to confirm the sequencing results, we used transmission electron microscopy (TEM). Morphological traits allow for viral identification up to the family or genus level [36]. Figure 4 displays sample images of *Hydra*-associated viruses as visualized by TEM. Viral families identified by this approach include the bacteriophages *Myoviridae*, *Siphoviridae*, *Podoviridae*, and *Inoviridae* (Figure 4 A–D), eukaryotic viral families *Herpesviridae* and *Phycodnaviridae* (Figure 4 E–F), as well as *Herpesviridae* and *Baculovirdae* viruses found associated with *Hydra* tissue (Figure 4 G–H). The viral morphotypes identified by ultrastructural analysis validates the virome families discovered by the molecular sequencing approaches, and thereby reassures us that we have uncovered the major components of the *Hydra* virome.

### Specific Species of Viruses Associating with *Hydra* Confirmed by Sequence Coverage

**Figure 5:**
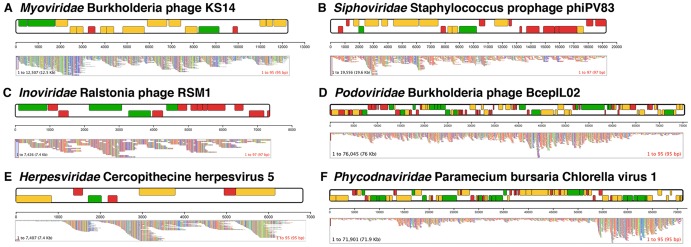
Assembled sequence coverage of prokaryotic and eukaryotic viruses from *Hydra* viromes. A) Assembled sequence of *Myoviridae* Burkholderia Phage KS14 from wild-caught *H. vulgaris* non-stressed virome. B) Assembled sequence of *Siphoviridae* Staphylococcus prophage phiPV83 from wild-caught *H. vulgaris* heat-stressed virome. C) Assembled sequence of *Inoviridae* Ralstonia Phage RSM1 from *H. vulgaris* (AEP) heat-stressed virome. D) Assembled sequence of *Podoviridae* Burkholderia phage BcepIL02 from wild-caught *H. vulgaris* non-stressed virome. E) Assembled sequence of *Herpesviridae* Cercopithecine Herpesvirus 5 from *H. vulgaris* (AEP) non-stressed virome. F) Assembled sequence of *Phycodnaviridae* Paramecium bursaria Chlorella Virus 1 from *H. viridissima* non-stressed virome. Each bar in the top part of each panel indicates an open reading frame from the viral sequence. Green bars indicate known gene function. Yellow bars indicate putative gene function. Red bars indicate unknown gene function. The bottom part of each panel indicates the depth of coverage for each assembled viral sequence.

To further reveal the constituents of the *Hydra* virome at the viral species level, we identified *Hydra* associated viral species through *de novo* sequence assembly. Figure 5 displays the assembly of representative viral species associating with *Hydra*. Examples of six assemblies are shown, reflecting the families of viruses identified earlier by TEM. *Myoviridae* Burkholderia KS14 sequence was assembled from the non-stressed wild-caught *H. vulgaris* virome (Figure 5A), while *Siphoviridae Staphylococcus* prophage phiPV83 sequence was assembled from the heat-stressed wild-caught *H. vulgaris* sample (Figure 5B). Since the latter is a prophage, the presence of this assembled genome confirms that environmental heat-stress did cause temperate to lytic conversion. It should be noted that *Staphylococcus* is not normally associated with laboratory strains of *Hydra*, yet it may have been present in this *Hydra* sample isolated from the wild. *Inoviridae* Ralstonia phage RSM1 was assembled nearly in full from the heat-stressed *H. vulgaris* (AEP) sample (Figure 5C). This bacteriophage was the most common viral sequence in the sample and its near full-length assembled genome indicates that the virus may serve a role in the *Hydra*-associated microbiome (Table S1). Additionally, *Podoviridae* Burkholderia phage BcepIL02 sequence was also assembled from the wild-caught *H. vulgaris* non-stressed virome (Figure 5D). Commonly assembled eukaryotic viral species included the *Herpesviridae* Cercopithecine Herpesvirus 5 and the *Phycodnaviridae* Paramecium bursaria Chlorella virus 1 (Figure 5E–F). Assembly of Chlorella virus 1 from the *H. viridissima* non-stressed sample is intriguing as confirmation of the Van Etten group's finding of a similar virus from isolated *Hydra* Chlorella [37]. The presence of this virus through sequence assembly implicates the virus in symbiosis with the algal Chlorella within the host *H. viridissima*. Assembled viral species genomes and their percent coverage are shown in Table S1.

### *Hydra*-Associated Viruses Play a Role in the Metabolism of the Holobiont

**Figure 6:**
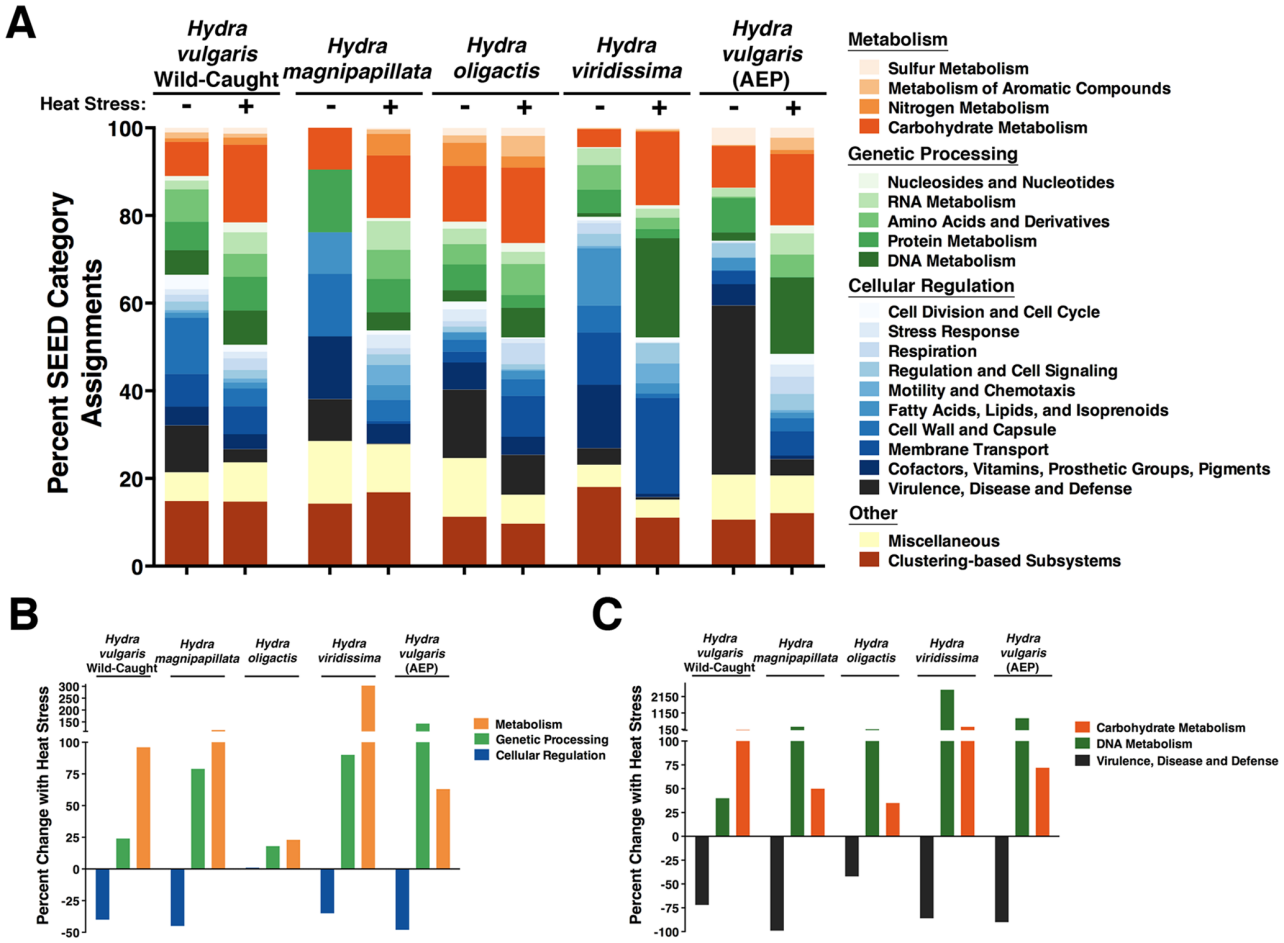
Effects of *Hydra*-associating viruses on metabolic subsystems. Relative abundances of sequences assigned to each cellular metabolic subsystem by MG-RAST. Assembled sequences were submitted to MG-RAST and TBLASTX was used to compare to a metabolic subsystem SEED database (E-value threshold ≤10^−5^). A) Values show the percent relative abundance of each SEED category assignment for each virome with respect to their effect on metabolism, genetic processing, and cellular regulation. The order of the subsystem in each column is equivalent to the order of the subsystem in the legend. B) Percent changes with heat-stress for each *Hydra* species of the three general groupings from (A). C) Percent changes with heat-stress for each *Hydra* species from three specific subsystems in (A).

Viruses are not just transient visitors within a host-associated environment, they are also involved in the metabolism of the associated microbial community [38]. Viruses can affect the associated cellular communities through their abilities to infect and transfer genes allowing for changes in the holobiont. To determine whether the *Hydra*-associated viruses have the potential to affect the metabolism of the holobiont, we compared the assembled viral sequences to a SEED metabolic subsystem database to determine the functional role of these viral genes on molecular pathways of specific biological processes [27]. Figure 6A shows the functional metabolic community profile of the viromes associating with each species of *Hydra*, indicating that many cellular functions in the holobiont are affected by the increase in viral diversity upon heat-stress. We used XIPE-Totec pairwise statistical comparison of the non-stressed versus heat-stressed conditions from each species to determine whether the shifts observed in the cellular metabolic and genetic processing subsystems were increased overall by heat-stress (Figure 6B) [28]. Comparatively, effects on cellular regulation subsystems were reduced. The increase of cellular metabolism and cellular genetic processing, as well as compromised cellular defense mechanisms, indicates viral involvement in the regulation of the host-associated microbiota under these conditions. Specifically, cellular DNA and carbohydrate metabolism subsystems were increased with heat-stress, while cellular virulence and defense mechanisms were nearly ablated upon heat-stress (Figure 6C). These data combine to illuminate the potentially dynamic effects of viruses on the metabolism of the holobiont under changing environmental conditions.

## Discussion

Until now it has been unclear whether viral communities are species-specific and whether these communities contribute to the multicellular holobiont. Using a metagenomic approach we analyzed five different *Hydra* species, including laboratory-housed animals and wild-caught animals, under non-stressed and environmentally stressed conditions, for a total of 10 viromes. This study suggests that each species of *Hydra* are associated with a diverse virome and virome composition is altered upon heat-stress. This virome change implies that the animals dynamically regulate their associated viral communities to compensate for changing environmental factors. Our results show that there are primarily bacteriophages associating with *Hydra*, that the *Herpesviridae* family of viruses predominate the eukaryotic viral population, and provides the possibility that viruses associated with *Hydra* affect the metabolism of the holobiont system. These data support the possibility that the host *Hydra* selectively shapes its virome and suggests that genetic factors of the host can outweigh environmental influences in determining viral colonization. The genetic forces of *Hydra* that shape the virome composition remain to be uncovered.

*Hydra* have a species-specific virome that reflects the species-specific *Hydra* microbiota. The classes of predicted bacteriophage hosts appear to be similar to that of the classes of bacteria that have been reported to associate with *Hydra* as most *Hydra* species primarily harbor proteobacteria [7], [10]. The virome data predict that the obligate bacterial hosts for the bacteriophages associated with *Hydra* are proteobacteria, with large populations infecting γ- and β-proteobacteria hosts. Environmental heat-stress increases the diversity of predicted hosts for the bacteriophages indicating induction of the lysogenic cycle. This temperate lifestyle of associated bacteriophage is reminiscent of viromes isolated from the human gut where stability of the viral communities was observed over time more so than an active kill-the-winner marine environment virome with boom-bust cycles [39], [40], [41]. It also suggests the dynamic selection of the associated viruses at the epithelium by the host *Hydra*. *Hydra* may select for viruses to regulate the growth rates of associated prokaryotic and eukaryotic cells. Therefore, *Hydra*-mediated r- selection of small, high-virulence viruses to infect rapidly growing prokaryotic cells and K-selection of large, low-virulence viruses to infect slow growing cells keeps bacterial hosts at low abundance through strong *Hydra* and bacteriophage control [41], [42]. Lastly, the apparent species-specificity of the associated viromes agrees with the concept of phylosymbiosis [43], [44]. This concept postulates that hosts select for their associated microbiome and this selection drives speciation. We have observed this phenomenon previously in *Hydra* with species-specific bacterial community selection [7], [10]. We observe here phylosymbiotic *Hydra* species-specific viral community selection.

An important and interesting finding in this study is the large percentage of *Herpesviridae* family viruses associating with the various species of *Hydra*. These viruses are rarely found in water environments, and are often only found associated with animals [45], [46]. This indicates that there is not a random association of viruses occurring from the water, rather, it implies an active selection of viruses by the host *Hydra*. These viruses may be commonly associated with the Cnidarian phylum and therefore may confer a benefit to the holobiont [30]. Nowhere is this more evident than in the *H. vulgaris* animals caught from the wild. These animals associated with a large population of *Herpesviridae* viruses, accounting for a quarter of the total viral families, and seemed to be unaffected by heat-induced stress. Additionally, the finding of *Herpesviridae* family and species confirms an earlier report of its genomic presence in ESTs culled from the *Hydra* genome [30]. The large abundances of eukaryotic viruses, particularly vertebrate-infecting viral families, are different from that of other invertebrate animals, specifically *D. melanogaster* and *C. elegans*, whose reported viruses are primarily invertebrate infecting ones [47], [48], [49]. Our data agree with earlier observations that Cnidaria have retained many ancestral genes that have been lost in *D. melanogaster* and *C. elegans* and that the genome organization and genome content of *Hydra* is remarkably similar to that of vertebrates [50], [51].

We found very few plant or algal viruses in these viral metagenomes. This may in part be due to sequencing only DNA viruses, as many known plant and algal viruses are RNA viruses. Therefore, the plant and algal viral communities associating with *Hydra* may be further revealed with sequencing of the RNA viruses. Of note, however, is the partial assembly of Chlorella virus 1 (Figure 4F), an algal infecting virus. The sequence of this DNA virus is similar to previous findings by Van Etten, et al., who identified it as *Hydra Viridis* Chlorella Virus-1 (HVCV-1) [37], [52]. These previous findings, combined with the presence of an assembled Chlorella virus-1 in the *H. viridissima* virome, suggest that algal viruses are possible participants in the *H. viridissima-*associated viral communities. These findings further the concept of species-specific symbioses.

Lastly, we observed changes in the metabolic potential of the holobiont system upon environmental stress (Figure 6). Instead of using signature genes, such as 16S ribosomal RNA gene, the complete genetic information of the viral community in a metagenome allows one to determine the functional potential of each community. As mentioned from the data in Figure 6, there were increases in metabolism and genetic processing subsystems, which were accompanied by a reduction in cellular regulation subsystem pathways upon heat-stress in all five *Hydra* species analyzed. Since only viral genes were submitted in this analysis, we can omit the possibility that the host itself changed its metabolic potential and that viruses are affecting the changes observed. The presence of these genes in the viromes and the effects they have on cellular subsystems indicates the potential impact of viruses in the *Hydra*-associated holobiont.

Our findings that multicellular animal hosts are associated with a unique viral community helps to provide a new basis for understanding the role of viruses in animal evolution and extends the concept of phylosymbiosis to include viral involvement. An intriguing future question is the nature of the forces that shape the specific viromes. Many selective forces may influence the composition of virome within the holobiont, including nutrients, microbe-microbe interaction, pressures from the external environment and host-derived factors [53], [54]. Up to now, viruses were mostly considered as pathogenic agents. However, ideas about the role of viruses in multicellular organisms are changing, and might be undergoing a paradigm shift. Using the *Hydra* system and its experimental approachability will further our understanding of the impact of the virome on host physiology.

## Supporting Information

Figure S1 **Viral and bacterial enumeration by fluorescent microscopy.** The VLP to bacteria ratio (mean ± SEM); *H. vulgaris* (AEP) non-stressed 4.4±0.6; *H. vulgaris* (AEP) heat-stressed 2.3±0.3; *Hydra* media non-stressed 7.1±0.5; *Hydra* media heat-stressed 7.6±0.8. Unpaired Student's t-test was performed to evaluate statistical significance. NS indicates “not significant”, * indicates p<0.05, *** indicates p<0.0001. Results are cumulated from replicate experiments with>30 images taken from each experiment.
(JPG)

Figure S2 **Rarefaction analysis of** ***Hydra*** **viromes compare to similarly sequenced viromes.** MG-RAST rarefaction analysis of the *Hydra* viromes compared to published viromes using 454 sequencing. The analysis was conducted by comparing the number of sequences by the number of gene clusters. Coral and freshwater viromes were used as comparison.
(JPG)

Figure S3 ***Hydra*** **viromes compare to water associated animal and freshwater viromes.** Principal component analyses of the *Hydra* viromes with published viromes from animal samples (A) and from freshwater samples (B). The PCoA was run in MG-RAST against the SEED database to determine variations between the samples.
(JPG)

Table S1 **Assembled viral species from** ***Hydra*** **viromes.**
(DOCX)
