# Supplementary figures and images for: Species-Specific Viromes in the Ancestral Holobiont Hydra
Source: PLoS One. 2014 Oct 24;9(10):e109952. doi: 10.1371/journal.pone.0109952 (PMC4208763; doi:10.1371/journal.pone.0109952)

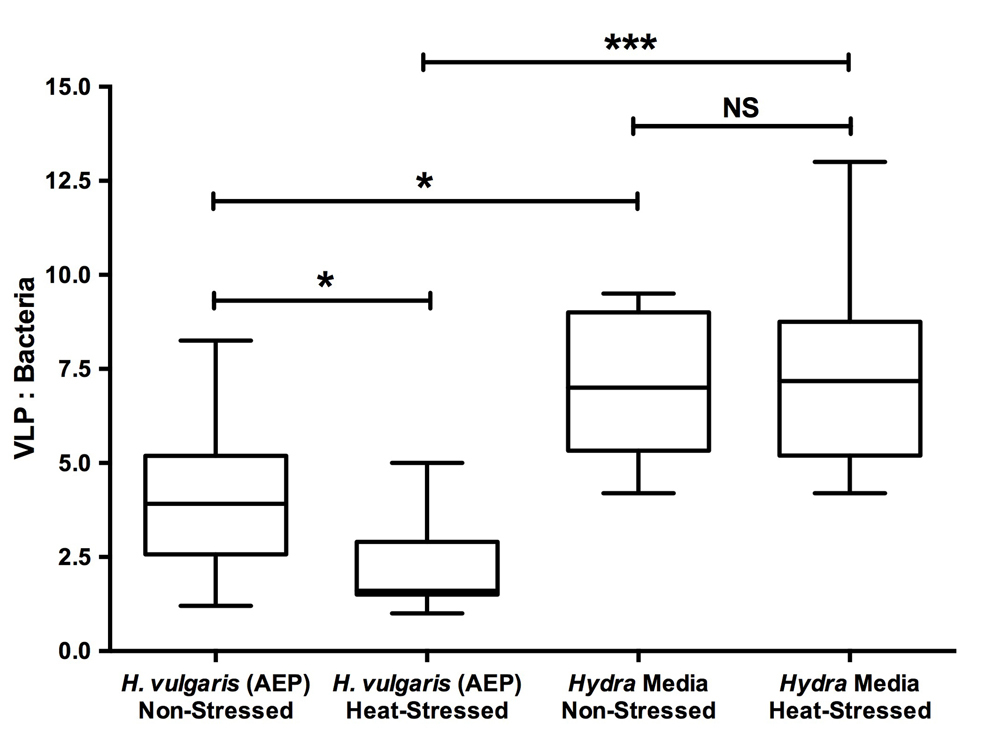

Supplement: Figure S1 — Viral and bacterial enumeration by fluorescent microscopy. The VLP to bacteria ratio (mean ± SEM); H. vulgaris (AEP) non-stressed 4.4±0.6; H. vulgaris (AEP) heat-stressed 2.3±0.3; Hydra media non-stressed 7.1±0.5; Hydra media heat-stressed 7.6±0.8. Unpaired Student's t-test was performed to evaluate statistical significance. NS indicates “not significant”, * indicates p<0.05, *** indicates p<0.0001. Results are cumulated from replicate experiments with>30 images taken from each experiment. (JPG) [file pone.0109952.s001.jpg]

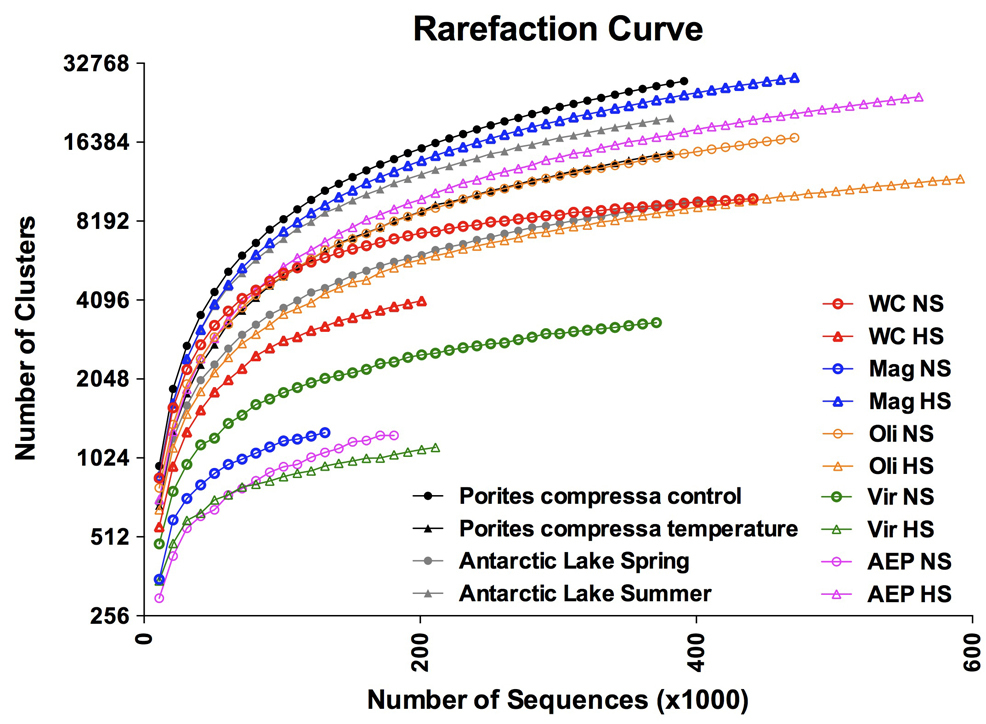

Supplement: Figure S2 — Rarefaction analysis of Hydra viromes compare to similarly sequenced viromes. MG-RAST rarefaction analysis of the Hydra viromes compared to published viromes using 454 sequencing. The analysis was conducted by comparing the number of sequences by the number of gene clusters. Coral and freshwater viromes were used as comparison. (JPG) [file pone.0109952.s002.jpg]

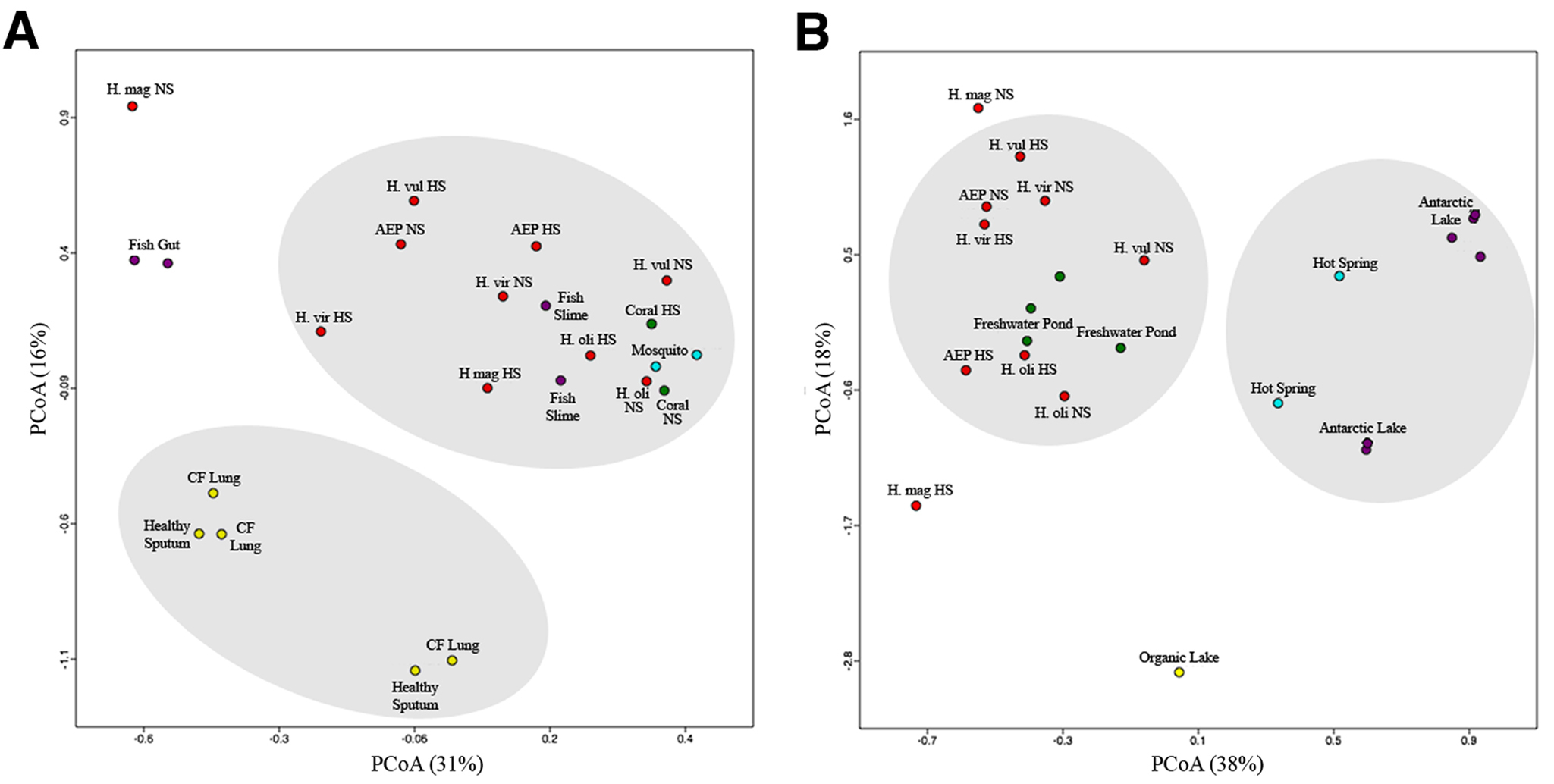

Supplement: Figure S3 — Hydra viromes compare to water associated animal and freshwater viromes. Principal component analyses of the Hydra viromes with published viromes from animal samples (A) and from freshwater samples (B). The PCoA was run in MG-RAST against the SEED database to determine variations between the samples. (JPG) [file pone.0109952.s003.jpg]
