# Supplementary material for: Species-Specific Viromes in the Ancestral Holobiont Hydra
Source: PLoS One. 2014 Oct 24;9(10):e109952. doi: 10.1371/journal.pone.0109952 (PMC4208763; doi:10.1371/journal.pone.0109952)
Supplement: Table S1 — Assembled viral species from Hydra viromes. (DOCX) [file pone.0109952.s004.docx]

| Hydra | Treatment | Viral Family | Viral Species | Viral Species ID | Type | Topology | Assembled Genome Coverage |
| --- | --- | --- | --- | --- | --- | --- | --- |
| *H. vulgaris*  Wild-Caught | Non-Stressed | *Inoviridae* | Ralstonia phage RSM3 | NC_011399.1 | ssDNA | Circular | 83.23% |
|  |  | *Myoviridae* | Burkholderia phage KS14 | NC_015273.1 | dsDNA | Circular | 46.15% |
|  |  |  | Clostridium phage phiCD27 | NC_011398.1 | dsDNA | Linear | 27.04% |
|  |  | *Phycodnaviridae* | Paramecium bursaria Chlorella virus 1 | NC_000852.5 | dsDNA | Linear | 21.52% |
|  |  | *Podoviridae* | Burkholderia phage BcepIL02 | NC_012743.2 | dsDNA | Linear | 90.15% |
|  |  |  | Planktothrix phage PaV-LD | NC_016564.1 | dsDNA | Linear | 19.58% |
|  |  | *Siphoviridae* | Microbacterium phage Min1 | NC_009603.1 | dsDNA | Linear | 70.97% |
|  |  |  | Stx2-converting phage 1717 | NC_011357.1 | dsDNA | Linear | 22.00% |
|  | Heat-Stressed | *Myoviridae* | Mycobacterium phage Cali | NC_011271.1 | dsDNA | Circular | 5.74% |
|  |  | *Podoviridae* | Planktothrix phage PaV-LD | NC_016564.1 | dsDNA | Linear | 21.31% |
|  |  | *Siphoviridae* | Enterobacteria phage BP-4795 | NC_004813.1 | dsDNA | Linear | 51.98% |
|  |  |  | Microbacterium phage Min1 | NC_009603.1 | dsDNA | Linear | 1.50% |
|  |  |  | Staphylococcus prophage phiPV83 | NC_002486.1 | dsDNA | Linear | 54.92% |
|  |  |  | Stenotrophomonas phage S1 | NC_011589.1 | dsDNA | Linear | 8.00% |
| *H. oligactis* | Non-Stressed | *Inoviridae* | Ralstonia phage RSM1 | NC_008574.1 | ssDNA | Circular | 95.10% |
|  |  |  | Ralstonia phage RSM3 | NC_011399.1 | ssDNA | Circular | 22.00% |
|  |  | *Phycodnaviridae* | Ostreococcus Lucimarinus Virus OLV1 | NC_014766.1 | dsDNA | Linear | 12.75% |
|  |  | *Podoviridae* | Burkholderia phage BcepIL02 | NC_012743.2 | dsDNA | Linear | 2.46% |
|  |  | *Siphoviridae* | Burkholderia phage BcepGomr | NC_009447.1 | dsDNA | Linear | 24.95% |
|  |  |  | Clostridium phage phiCTP1 | NC_014457.1 | dsDNA | Linear | 33.08% |
|  |  |  | Stenotrophomonas phage S1 | NC_011589.1 | dsDNA | Linear | 26.45% |
|  | Heat-Stressed | *Inoviridae* | Ralstonia phage p12J | NC_005131.2 | dsDNA | Circular | 91.77% |
|  |  | *Myoviridae* | Aeromonas phage Aeh1 | NC_005260.1 | dsDNA | Linear | 2.45% |
|  |  |  | Aeromonas phage phiAS5 | NC_014636.1 | dsDNA | Linear | 5.20% |
|  |  | *Phycodnaviridae* | Paramecium bursaria Chlorella virus 1 | NC_000852.5 | dsDNA | Linear | 5.76% |
|  |  | *Siphoviridae* | Burkholderia phage BcepGomr | NC_009447.1 | dsDNA | Linear | 17.46% |
|  |  |  | Burkholderia phage phiE125 | NC_003309.1 | dsDNA | Linear | 25.77% |
| *H. magnipapillata* | Heat-Stressed | *Inoviridae* | Ralstonia phage p12J | NC_005131.2 | dsDNA | Circular | 90.55% |
|  |  |  | Ralstonia phage RSM1 | NC_008574.1 | ssDNA | Circular | 96.92% |
|  |  | *Iridoviridae* | Lymphocytis disease virus - Isolate China | NC_005902.1 | dsDNA | Linear | 11.70% |
|  |  | *Polydnaviridae* | Cotesia congregata bracovirus | NC_006658.1 | dsDNA | Circular | 5.80% |
| *H. viridissima* | Non-Stressed | *Inoviridae* | Ralstonia phage RSM3 | NC_011399.1 | ssDNA | Circular | 32.72% |
|  |  | *Myoviridae* | Burkholderia phage KS14 | NC_015273.1 | dsDNA | Circular | 90.22% |
|  |  |  | Salmonella phage Fels-2 | NC_010463.1 | dsDNA | Linear | 24.22% |
|  |  | *Phycodnaviridae* | Paramecium bursaria Chlorella virus AR158 | NC_009899.1 | dsDNA | Linear | 3.54% |
|  |  | *Podoviridae* | Planktothrix phage PaV-LD | NC_016564.1 | dsDNA | Linear | 6.45% |
|  |  | *Siphoviridae* | Enterobacteria phage BP-4795 | NC_004813.1 | dsDNA | Linear | 3.42% |
|  |  |  | Microbacterium phage Min1 | NC_009603.1 | dsDNA | Linear | 2.03% |
|  | Heat-Stressed | *Inoviridae* | Ralstonia phage RSM3 | NC_011399.1 | ssDNA | Circular | 36.31% |
|  |  | *Myoviridae* | Burkholderia phage KS14 | NC_015273.1 | dsDNA | Circular | 39.00% |
|  |  | *Podoviridae* | Enterobacteria phage Phieco32 | NC_010324.1 | dsDNA | Linear | 5.93% |
|  |  | *Siphoviridae* | Burkholderia phage phiE125 | NC_003309.1 | dsDNA | Linear | 7.30% |
|  |  |  | Enterobacteria phage BP-4795 | NC_004813.1 | dsDNA | Linear | 1.53% |
| *H. vulgaris* (AEP) | Non-Stressed | *Herpesviridae* | Cercopithecine herpesvirus 5 | NC_012783.2 | dsDNA | Linear | 10.03% |
|  | Heat-Stressed | *Inoviridae* | Ralstonia phage RSM1 | NC_008574.1 | ssDNA | Circular | 81.76% |
